# The integrated stress response pathway and neuromodulator signaling in the brain: lessons learned from dystonia

**DOI:** 10.1172/JCI177833

**Published:** 2024-04-01

**Authors:** Nicole Calakos, Zachary F. Caffall

**Affiliations:** 1 Department of Neurology,; 2 Department of Neurobiology, and; 3 Department of Cell Biology, Duke University Medical Center, Durham, North Carolina, USA.; 4 Aligning Science Across Parkinson’s (ASAP) Collaborative Research Network, Chevy Chase, Maryland, USA.

## Abstract

The integrated stress response (ISR) is a highly conserved biochemical pathway involved in maintaining proteostasis and cell health in the face of diverse stressors. In this Review, we discuss a relatively noncanonical role for the ISR in neuromodulatory neurons and its implications for synaptic plasticity, learning, and memory. Beyond its roles in stress response, the ISR has been extensively studied in the brain, where it potently influences learning and memory, and the process of synaptic plasticity, which is a substrate for adaptive behavior. Recent findings demonstrate that some neuromodulatory neuron types engage the ISR in an “always-on” mode, rather than the more canonical “on-demand” response to transient perturbations. Atypical demand for the ISR in neuromodulatory neurons introduces an additional mechanism to consider when investigating ISR effects on synaptic plasticity, learning, and memory. This basic science discovery emerged from a consideration of how the ISR might be contributing to human disease. To highlight how, in scientific discovery, the route from starting point to outcomes can often be circuitous and full of surprise, we begin by describing our group’s initial introduction to the ISR, which arose from a desire to understand causes for a rare movement disorder, dystonia. Ultimately, the unexpected connection led to a deeper understanding of its fundamental role in the biology of neuromodulatory neurons, learning, and memory.

In this Review, we focus on the integrated stress response (ISR), a broadly conserved biochemical pathway that regulates proteostasis in nearly all cell types. The ability to maintain cellular homeostasis in the face of diverse insults is a requirement for cell survival and evolutionary fitness. A number of cell stress response and proteostasis pathways have evolved to meet this need. Elements of these pathways are widely shared across diverse evolutionary phyla. In addition, within a multicellular organism, the same core biochemical pathways appear to be active in all cell types, despite varying cellular demands and physiology. Thus, a key feature of such cell stress and proteostasis pathways is a remarkable dynamic range in the ability to recognize deviations from steady state and trigger a response. In view of the pervasive importance of cell stress responses across cell and tissue types, a quandary presents itself when one considers the relative specificity of clinical syndromes that arise in genetic disorders involving proteostasis pathway components.

Dystonia is among the latest clinical disorders to be recognized as an ISR disorder (1, 2). Dystonia refers to the presence of involuntary muscle movements that present as slow twisting movements and abnormal posture (1). The movements often involve abnormal co-contraction of agonist and antagonist muscle pairs and are driven by central nervous system dysfunction. While a range of clinical presentations and contexts include dystonia, a peculiar feature of a subset known as “task-specific dystonias” is that involuntary movement is only triggered by a specific learned skill. For instance, difficulty controlling the hand occurs when performing a specific action, such as playing the violin or writing, and the hand can function normally in other use settings (3, 4). This particular clinical feature was the hook that motivated our laboratory to determine its cause, as our overarching interests are to understand how the brain learns and adapts to experience, and how and when these processes go awry in disease.

In searching for this answer over the last decade, we learned about the causes of dystonia. But on top of this, in struggling with the quandary of why impairment of a remarkably generalized cellular process led to such a specific clinical disease, we gained new insights into how the ISR is used in the healthy brain. Certain brain cell subtypes appear to engage stress response pathways basally for their everyday function. In the brain, basal ganglia cells releasing the neuromodulatory neurotransmitter acetylcholine chronically activate the ISR in the normal healthy state, and manipulations that reduce this basally high level of ISR activation alter learning and memory (5). In the following discussion, we first introduce the ISR and its connection to dystonia and then discuss the ramifications that ISR activity in neuromodulatory neurons has for learning, memory, and synaptic plasticity. We chose these particular scientific contributions because the process of their discovery highlights the often unpredictable route from initial question to impact and the critical need to support a broad base of high-risk, curiosity-driven research, whether clinical or basic in origin.

## The ISR: a phospho-switch to change the proteome

**Figure 1:**
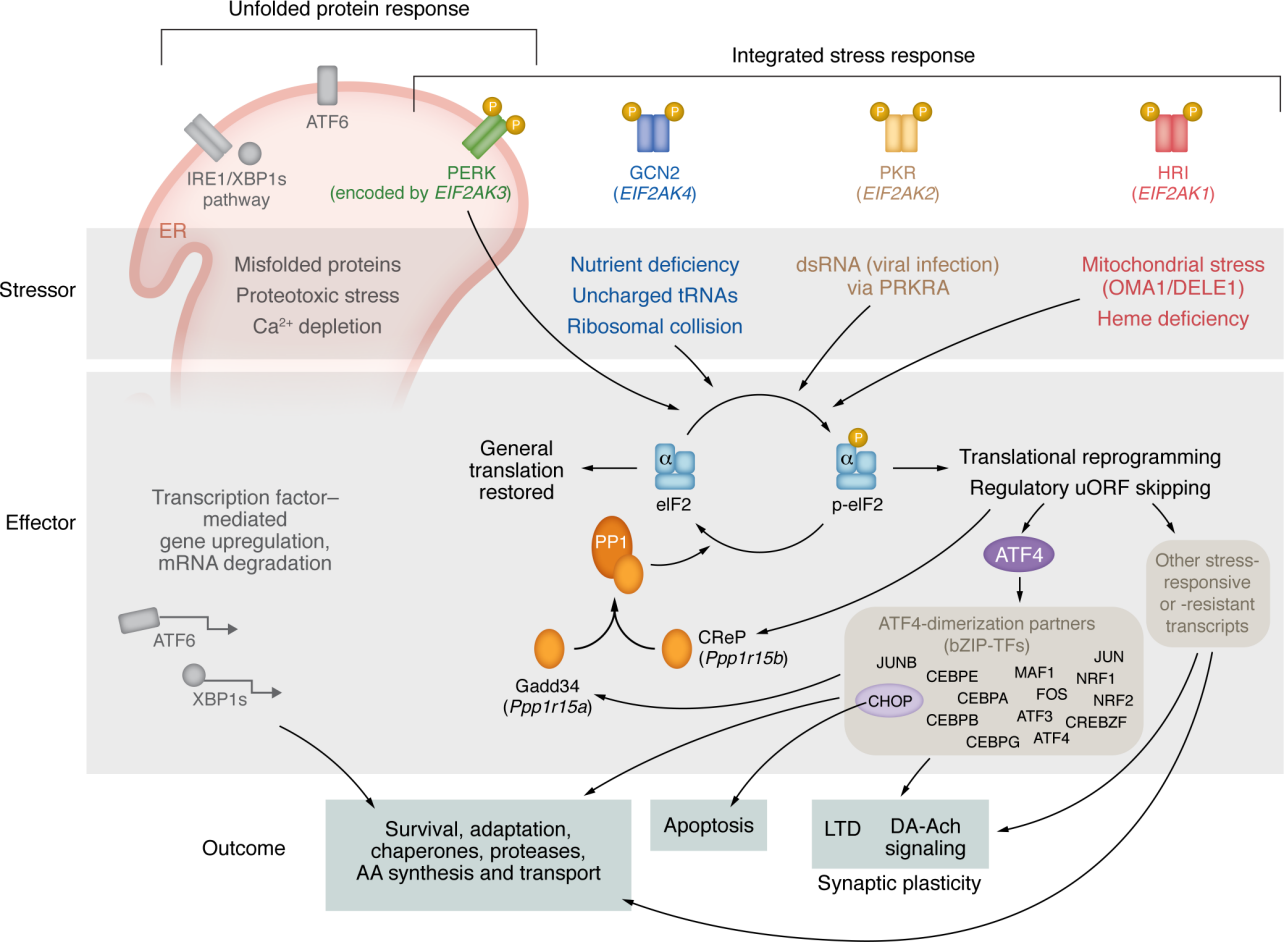
ISR pathway and outcomes. The ISR and its intersection, via PERK, with the UPR (upper left) constitute the 2 major proteostasis pathways. Members of the EIF2α kinase family respond to proteotoxic stress in the ER (PERK), nutrient deficiency in the cytosol (GCN2), viral infections (PKR), and oxidative stress arising from mitochondrial dysfunction (HRI). These 4 kinases share the same substrate, the α subunit of the trimeric translation initiation factor eIF2. Phosphorylation of eIF2α induces broad reprogramming of translation within the cell, preferentially translating mRNAs with certain regulatory upstream open reading frames (uORFs) and broadly reducing the translation of mRNAs without uORFs (6, 7). In addition to marked reduction in total protein translation, this action reduces cellular stress through adaption mediated though preferential translation and transcription of chaperones, proteases, and amino acid synthesis and transport proteins (7). The ISR is kept in check by 2 dedicated phosphatases, CReP and Gadd34, which dephosphorylate eIF2α, either constitutively (CReP) or as a part of the adaptive response to ISR pathway activation (Gadd34). Activating transcription factor 4 (ATF4) is the best-characterized effector of the ISR and promotes diverse outcomes dependent on the duration of the pathway activation and the presence of diverse dimerization partners that determine the broader impact of ATF4 transcriptional activity (93). Prolonged ISR pathway activation may exceed the adaptive response of the cell and lead to controlled cell death via apoptosis. In the CNS, ISR pathway activation has roles beyond response to cellular stresses. In the brain, ISR signaling influences synaptic plasticity, such as long-term depression (LTD) and neuromodulator signaling involving dopamine (DA) and acetylcholine (Ach) (12, 65, 76, 79).

The ISR refers to a highly conserved biochemical pathway that exerts broad control over which proteins are synthesized based on the phosphorylation status of the translation initiation factor eIF2α (eukaryotic initiation factor 2 α; encoded by *EIF2A*, also known as *EIF2S1*) (reviewed in ref. 6). EIF2α phosphorylation is known to be regulated by 4 kinases and 2 phosphatases: heme-regulated inhibitor (HRI, encoded by *EIF2AK1*); protein kinase R, interferon-inducible double-stranded RNA dependent (PKR, encoded by *EIF2AK2*); PKR-like ER kinase (PERK, encoded by *EIF2AK3*); general control nonderepressible 2 (GCN2, encoded by *EIF2AK4*); and protein phosphatase 1 (PP1, encoded by *PPP1CA*), with either of the following regulatory subunits — growth arrest and DNA damage–inducible 34 (GADD34, encoded by *PPP1R15A*) and constitutive regulator of eIF2α phosphorylation (CReP, encoded by *PPP1R15B*). The term “integrated stress response” (ISR) refers to the role of this pathway in maintaining cellular proteostasis in response to diverse cellular stressors (Figure 1). For example, PERK monitors protein misfolding and proteotoxic stress in the ER; GCN2 monitors nutrient deficiencies such as free amino acids and uncharged transfer RNAs (tRNAs); HRI monitors deficiencies in heme abundance often due to mitochondrial dysfunction; and PKR monitors double-stranded RNA/viral infections.

The ISR is considered active when eIF2α has been phosphorylated by one of the 4 eIF2α kinases. This phosphorylation of the α subunit of the eIF2 complex inhibits GDP-to-GTP exchange by eIF2B, slowing the rate of at which eIF2 can bind free initiator methionine tRNA, thereby slowing formation of the ternary complex that is needed to initiate translation. In this way, the kinetics of eIF2 complex recharging and loading limits the availability of the ternary complex to the scanning ribosome and delays reinitiation following regulatory upstream open reading frames (uORFs). Consequences of this are that proteins with certain regulatory uORF structures are preferentially translated and result in a reprogramming of the translatome dependent on mRNA structure. A number of comprehensive reviews describe these mechanisms in more detail (6, 7). In general, the consequence of eIF2α phosphorylation is a dramatic reduction in bulk protein synthesis and an increase in translation of a select subset of proteins tuned for adaption to the perceived stress (chaperones, proteases, amino acid synthesis, and transport proteins). Among the proteins whose translation is increased are effectors such as the transcription factor ATF4 (also known as CREB2). While adaptation through upregulation of ER chaperones and antioxidant proteins combined with the reduction of the general protein translation burden is one outcome of the ISR, persistent activation can lead to CHOP- (or DDIT3-) mediated apoptosis.

**Figure 2:**
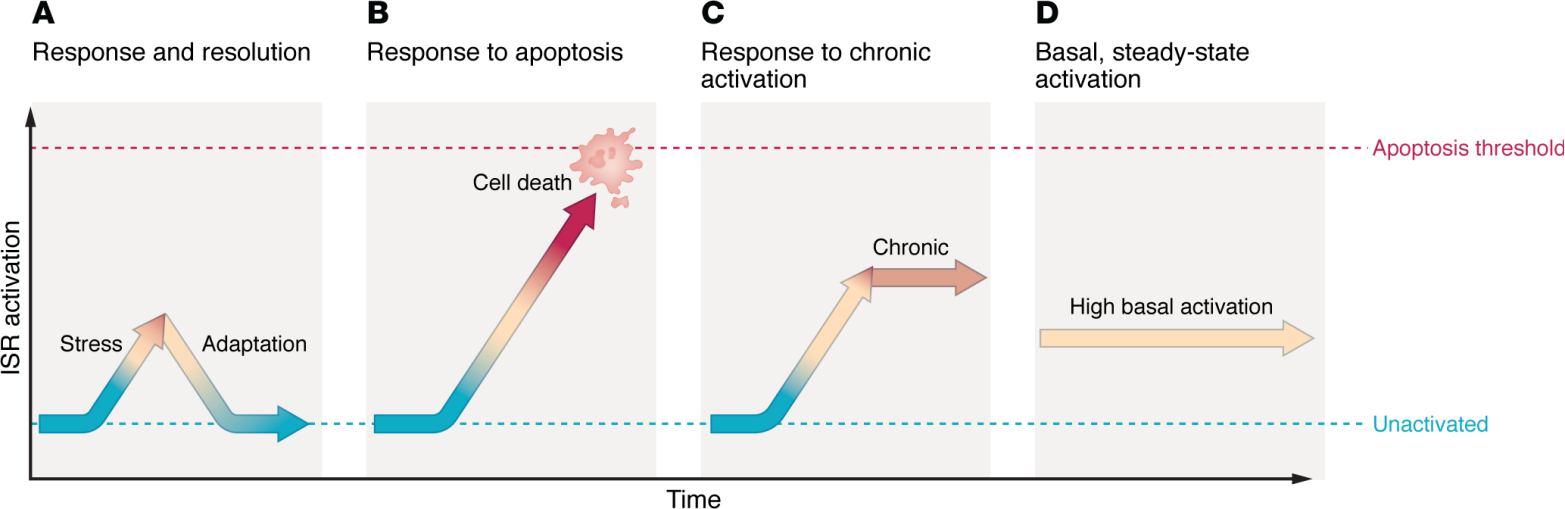
Modes of ISR activation. (**A**) Activation and adaptive resolution, by either cell stress or other experience, e.g., learning. (**B**) Excessive activation leading to apoptosis. (**C**) Activation followed by chronic activation, as in settings of chronic disease pathology. (**D**) Sustained activation in normal cells, as recently exemplified by striatal CINs (76).

The canonical view is that the ISR is conditionally activated by a cell stressor or perturbation (6, 8–10) (Figure 1 and Figure 2, A–C). Thereafter, ATF4-dependent transcriptional processes lead to either successful restoration of cellular homeostasis or CHOP-dependent apoptosis (Figure 1 and Figure 2B). As with any protein or pathway named for a particular cellular role, it is also possible that the core biochemical process may be adapted in specific cells or settings to have additional roles. For example, in the brain, the ISR plays additional roles in circadian rhythms, developmental axonogenesis, synaptic plasticity, learning, and memory (8, 11–13). For this reason, it is valuable to also conceptualize the ISR in its most generic form: as a protein phosphorylation–regulated process that causes major shifts in the proteome by influencing which reading frames are translated on messenger RNAs.

## ISR in brain disease: overview

It is not surprising that as a cellular pathway that plays a central role in responding to diverse cell stressors and restoring homeostasis, the ISR has been implicated in contributing to a wide range of disease processes. In brain disease, the ISR is often implicated as a disease accelerator when activated chronically under cell stressors associated with neurodegenerative diseases and aging (Figure 2C). In the brain, many proteotoxic and environmental stressors, as well as their accumulation with aging, are sufficient to activate the ISR. Given that the ISR is suitably poised to respond to diverse cell stressors — from misfolded proteins and oxidative stress to heme deficiency and viral infection — this pathway has been explored as a therapeutic target in many diseases (reviewed in refs. 9, 14). In principle, a finding of ISR activation in disease settings may signify roles as an exacerbator, compensator, or root cause, or may be completely incidental. However, while the ISR is implicated in many diseases by its activation, additional evidence to support the role that the ISR plays in the disease process is often lacking. One opportunity to better understand roles of the ISR in the brain is to invert the question and ask what consequences arise from ISR pathway hyper- or hypoactivity in the context of genetic diseases involving genes in the ISR pathway. Below, we discuss one recent example. An overview of genetic syndromes associated with the ISR can be found in ref. 15.

## ISR pathway dysfunction as a cause for dystonia

While the ISR may be activated to ameliorate, and possibly accelerate, pathobiology in diverse settings, in dystonia, convergent human genetics alongside functional experiments in model systems point to disruptions of ISR pathway function as a causal mechanism for disease. Dystonia is a movement disorder characterized by loss of voluntary control over movement, presenting as slow twisting movements and abnormal sustained postures. The first inroad to understanding molecular mechanisms for the disorder came in 1997 with the discovery of the gene mutation responsible for a monogenic inherited form of dystonia originally called DYT1 and now referred to as DYT-TOR1A (16). The DYT1-causing gene, *TOR1A*, encodes the Torsin1a protein, which is a member of the AAA+ ATPase family of proteins. Because the AAA+ ATPase family includes proteins with chaperone function, the effects of the DYT1-causative *TOR1A* mutation on cell stress responses were examined by a number of laboratories. Using diverse assays, multiple groups identified abnormalities in readouts of cell stress responses, from accumulation of misfolded proteins to increased chaperone production in the ER (17–20). With recent human genetics and functional genomics more specifically pointing to perturbations in the ISR as causing dystonia, a potential mechanism for these earlier findings may reside in the crosstalk between the unfolded protein response (UPR) and the ISR via PERK activation (Figure 1).

**Figure 3:**
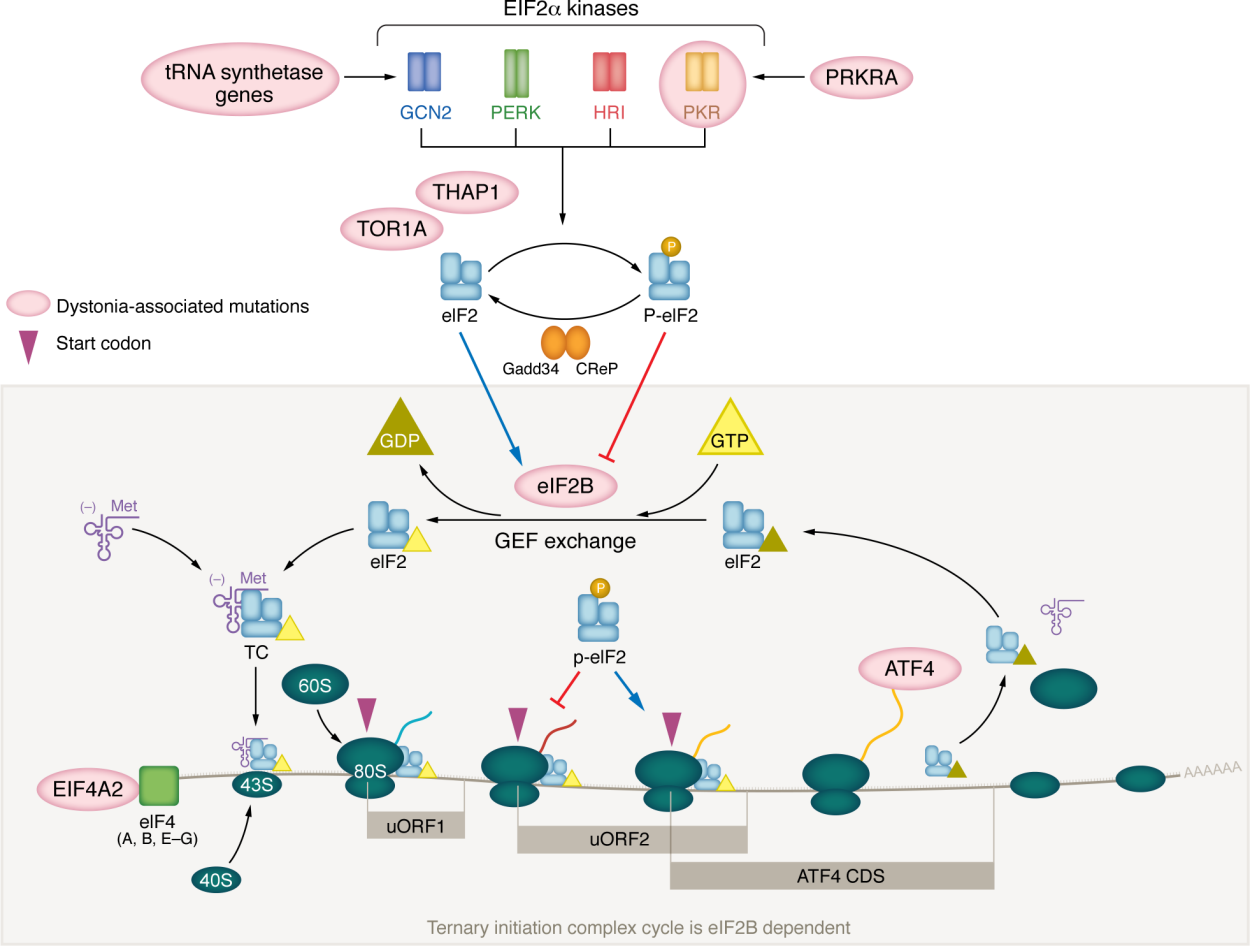
Dystonia-associated mutations in genes influencing the activation and outcome of the ISR. A summary of the ISR pathway’s relationship to key components of translational initiation highlights intersections with proteins encoded by genes harboring mutations associated with clinical phenotypes of dystonia. Functional evidence supports ISR involvement in multiple monogenic forms of dystonia and dystonia/Parkinsonism (*PRKRA* [refs. 21, 22, 94], *TOR1A* [refs. 5, 29], *THAP1* [ref. 32]). Human genes associated with effects on the ISR, or translational initiation, that present with clinical phenotypes of dystonia include PKR (*EIF2AK2*) (23–27), *EIF2B* (42, 95), *ATF4* (5), *EIF4A2* (28), and a range of tRNA synthetase genes associated with mitochondrial disorders (33–41) (e.g., *AARS1*, *AARS2*, *CARS2*, *EARS2*, *WARS2*, *TARS2*). Activation of the ISR results in phosphorylation of the α subunit of eIF2 and reduces the rate of eIF2B-mediated GDP/GTP exchange of eIF2, preventing the formation of the ternary complex (TC) (GTP-eIF2-Met tRNA). Reduction in TC abundance limits the rate of elongation reinitiation following regulatory upstream open reading frames (uORFs) by slowing the rate of preinitiation complex (PIC; “43S”) formation (6, 7, 9). This delay in reinitiation following a regulatory uORF results in translational reprogramming based on mRNA uORF structure. ATF4 is preferentially translated under these conditions and is the best-characterized effector of ISR pathway activation. As it is the obligate guanine exchange factor for eIF2, formation of the TC is dependent on eIF2B function. EIF4A modifies mRNA secondary structure to enhance small ribosomal subunit (40S) mRNA scanning (96). Additionally, mutations in tRNA synthetase genes are known to chronically activate the ISR through stimulation of GCN2 (97) and may additionally delay translational initiation by limiting the availability of charged tRNAs.

In 2008, the ISR branch of cell stress responses per se (Figure 1) became implicated in dystonia when the cause for another monogenic form of dystonia and parkinsonism was associated with missense mutations in *PRKRA*, whose product PACT influences ISR activation (21) (Figure 3). Functional studies of the causative missense mutation showed that when ISR activation was evoked by a cell stressor, activation was blunted in the early phase and remained activated for a longer period of time (22). In 2016, similar ISR disruptions were identified in two additional dystonias (5). In that study, DYT-TOR1A patient–derived fibroblasts showed a blunted response to ISR activation evoked by the cell stressor thapsigargin, an effect attributed to the finding that at steady state, levels of the eIF2α phosphatase regulatory subunit CReP were abnormally elevated (5). In a sporadic form of focal dystonia, cervical dystonia, rare coding variants were enriched in the *ATF4* gene (note that although this study included a replication cohort, this association has not been confirmed by independent groups) (5). Functional studies showed that the dystonia-enriched variants reduced ATF4 transcriptional activity (5). The convergent findings of ISR dysfunction across 3 forms of dystonia was pivotal to supporting a causal role for the ISR and led to the hypothesis that weakened ISR activation is a shared pathway mechanism for dystonia (5). A subsequent human genetic association of dystonia with mutations in the *EIF2AK2* gene, which encodes the PKR kinase (23–27), further established the link between dystonia and the ISR. While a recent association of dystonia with mutation in the *EIF4A2* gene (28) highlights the functional intersection between dystonia and the translational initiation process. The encoded protein, eIF4A-2, is necessary to coordinate complexing of the mRNA with the eIF2 complex–associated, methionine tRNA–charged 43S ribosome. Additionally, evidence of ISR dysregulation using biochemical assessments was identified in multiple other dystonia model systems related to DYT-TOR1A, DYT-THAP1, and DYT-SCGE (29–32).

The association of ISR dysregulation with the clinical phenotype of dystonia warrants a closer reexamination for the presence of dystonia in clinical syndromes involving ISR or “ISR-adjacent” genes (Figure 3). Dystonia is a clinical entity that can be difficult to diagnose accurately outside of subspecialty clinics, as individuals notoriously experience yearslong delays from presentation to diagnosis. Diagnosis can be even more challenging if coexisting in a syndromic disorder with cognition and other neurological function affected. Nonetheless, there are already a few examples suggesting that the contexts in which ISR impairment may lead to dystonia may be considerably broader. For example, dystonia is a prominent feature of multiple genetic mitochondrial diseases involving tRNA synthetase genes (33–41). Since the ISR kinase GCN2 is activated by amino acid deficiencies that are sensed by the absence of loaded tRNAs, these disorders should impact the ISR as well. In vanishing white matter disease (VWMD) that is caused by mutation in the *EIF2B* gene, although the clinical presentation has not been well recognized as a cause for dystonia, the clinical spectrum includes at least some individuals who present with dystonia (42).

Additionally, evidence from cell and animal model systems supports that the directionality of ISR perturbation for the VWMD mutation causes excess, chronic ISR activation (43–45). Accordingly, VWMD is rescued by the ISR-inhibiting tool compound ISRIB and the eIF2B activator 2BAct (46, 47), whereas cell and animal model phenotypes of DYT1 expressing the *TOR1A* mutation are rescued by ISR-boosting compounds (salubrinal, Sal-003, ritonavir) (5). These data indicate that perturbations of ISR activity in either direction are associated with the dystonia phenotype. This situation is reminiscent of other disorders involving protein synthesis regulation, such as autism (48, 49). Among autism-related disorders, there are examples of mutational effects that cause increased or decreased protein synthesis, leading to concepts that translational regulation may need to be “just right” and not skewed in either direction. This may also be the case for ISR regulation in the context of dystonia. Collectively, these examples support the ISR biochemical pathway as a potential therapeutic target for dystonia in diverse settings (Figure 3). The critical paths ahead, translationally, will be to establish which ISR-modifying targets are appropriate for which specific dystonias and to define when interventions are most effective.

Understanding the relationship among the ISR, genetic risk, and dystonia penetrance is an area for further investigation, with possible implications for the timing of therapeutic interventions. Many genetic dystonias show reduced penetrance — i.e., only a subset of individuals with the disease mutations will ever manifest the disease. Empirically, success in eliciting dystonia in certain rodent models has been gained through two-hit models in which genetic risk is combined with an experience or lesion (50). Could an ISR-activating stressor, experience, or exposure be a “second hit” required for genetic risk to lead to dystonia? Although it can be difficult to establish epidemiological relationships between fairly common events, such as respiratory infections, and disease onset, particularly in rare diseases, clinical observations in several dystonias have raised the idea supporting a second hit (reviewed in ref. 51). Often such second hits are also events that would be predicted to engage the ISR. Additionally, in a study of genetic modifiers of dystonia penetrance, ISR-related genes were among genes identified as having a potential effect (52). Mechanistic studies in animal models can further help test the role of ISR-activating experiences in precipitating dystonia.

## ISR intersections with cells and circuits in dystonia

There are also a number of outstanding questions to address to better understand how ISR dysfunction leads to dystonia. While the ISR is a clear culprit in dystonia pathogenesis, we still don’t know exactly which cells, circuits, and specific cellular dysfunctions are the mediators of ISR disruptions that lead to dystonia. For dystonia, in general, it has been challenging to establish a particular cell type or single circuit driving the pathophysiology. Compounding this issue is that many rodent models of human genetic disease with construct validity have proven to be insufficient to manifest dystonia phenotypically on their own without further manipulations. A consideration of the intersection between ISR roles and dystonia pathomechanisms may refine this search. Briefly, we highlight 3 sites implicated in dystonia pathogenesis that may also derive from ISR dysfunction.

Apart from any considerations of the ISR, recent studies emphasize the potential importance of oligodendrocytes as a key cell type that may be involved in dystonia pathogenesis (reviewed in ref. 53). White matter differences have been described in human brain imaging of DYT-TOR1A, DYT-THAP1, DYT-COL6A3, and sporadic forms of spasmodic dysphonia, as a few examples (54–57). Recent translational studies further provide mechanistic support for vulnerabilities of oligodendrocytes in dystonia caused by THAP1 mutations (58–60). Intriguingly, white matter involvement is also a prominent feature of several ISR-related genetic disorders, centrally (e.g., mitochondrial diseases and the leukodystrophy VWMD) and/or peripherally (e.g., neuropathies such as Charcot-Marie-Tooth disease that are associated with tRNA synthetase gene mutations). And finally, there is some evidence to support the possibility that white matter abnormalities in dystonia are mechanistically related to ISR dysfunction. The white matter abnormalities in the DYT-TOR1A mouse model were corrected by treatment with an ISR-activating drug, ritonavir (61).

Another circuit vulnerability potentially leading to dystonia involves long-term synaptic plasticity in corticostriatal circuitry. Clinical aspects of certain dystonias have long been recognized as suggesting a potential underlying mechanism involving dysfunctional synaptic plasticity and motor learning (62). In support of this, the DYT-TOR1A mouse model shows excessive long-term potentiation (LTP) and deficits in an mGluR5-dependent form of long-term synaptic depression (LTD) at corticostriatal synapses (63). Outside of corticostriatal circuits, ISR inhibition has shown similar effects of shifting the neuronal activity thresholds needed to induce synaptic plasticity to favor LTP and disfavor LTD (64). Moreover, in the hippocampus, ISR activity has been shown to be necessary and sufficient to induce an mGluR5-dependent form of LTD (65). Subsequent tests of corticostriatal mGluR-LTD on striatal projection neurons support a role for the ISR. Inhibition of the ISR with ISRIB blocked LTD in WT mice, while augmentation of the ISR pathway using the phosphatase inhibitor Sal-003 rescued mGluR-LTD in the DYT-TOR1A mouse model (5).

Striatal cholinergic interneuron (CIN) dysfunction is another feature shared by several genetic dystonia mouse models. Additionally, CIN dysfunction may underlie the corticostriatal plasticity deficits as well. CINs play a facilitatory role for corticostriatal mGluR-LTD (66, 67), and anticholinergic compounds rescue dystonia model synaptic plasticity deficits (68). In dystonia models, striatal CINs show an inversion of the usual response to dopamine (often referred to as a “paradoxical” response). In response to dopamine signaling through type 2 dopamine receptors (D2Rs), CINs typically slow or pause their tonic firing rates. In contrast, in dystonia mouse models, CINs increase their firing rates with D2R agonism (69–72). This hyperactive CIN firing response is posited to explain the clinical effectiveness that anticholinergic drugs show for dystonia (72). In studies of the normal brain, this cell type stood out by showing the relatively unusual property of highly activating the ISR at steady state (73). In addition, inhibiting the ISR in CINs recapitulated the inverted physiological response to D2R agonism that characterizes dystonia mouse models (73).

These examples highlight the potential to use intersections between the ISR and dystonia to further establish the cell and circuit vulnerabilities that lead to dystonia. In the remainder of this Review, we more broadly consider the ramifications of steady-state ISR pathway engagement as a principle that governs striatal CIN neuromodulation, specifically with respect to the ISR’s role in learning and memory.

## ISR in neuromodulatory neurons influences learning and memory

Neuromodulatory neurotransmitters include chemical messengers such as acetylcholine, dopamine, and serotonin. They are distinguished from other neurotransmitters because they exert broad spatial effects (neurochemical signaling that diffuses beyond a single synapse, termed “volume transmission”) (74, 75). Neuromodulators markedly influence many aspects of circuit function, including the induction of synaptic plasticity; and behavior, particularly attention, learning, and memory (74, 75). The recognition that certain neuromodulatory neuron subclasses heavily activate the ISR at steady state in the normal brain was made using a newly developed approach to functionally interrogate the ISR.

Methods to measure ISR activity in the brain have been relatively limited due to the suboptimal properties of available immunohistochemical reagents and functional readouts that lack cellular-level resolution (76). The genetic reporter SPOTlight is a recently developed tool for functionally phenotyping ISR activity. SPOTlight provides cellular resolution readouts of translation initiation activity (73). In SPOTlight, the coding sequence for GFP is under the control of an upstream open reading frame (uORF2) that is typically used when eIF2α is unphosphorylated, whereas a red fluorescent protein (RFP) is in the main ORF that normally encodes Atf4 and is translated when eIF2α is phosphorylated. In this way, the ratio of RFP to GFP serves as a functional readout of the relative abundance of ISR-dependent translational activity in a cell.

Using SPOTlight, our group identified an unusual role for a population of CINs in engaging the ISR (73). Striatal CINs as a cell class engaged the ISR at normal baseline conditions, whereas most cells did not (73). The ISR is often conceptualized as a “response” pathway induced — typically transiently — by something such as cell stress or learning experiences or during specific developmental periods (6, 8–10) (Figure 2A). The example of striatal CINs expands the modes of ISR engagement to include chronic activation at steady state under normal, healthy conditions (Figure 2D). This diversity of ISR requirement by specific cell types suggests the need for a more nuanced view of what levels of ISR activation are associated with health.

In the brain, striatal CINs are among the few types of neurons that have autonomously driven, tonically active action potential firing. This feature, at least in part, drives ISR activation in CINs, since chronic inhibition of these neurons (by a G_i/o_ DREADD) lowered their level of ISR activation (73). However, it does not appear that this ISR engagement feature is present in all cells with tonic or high-firing activity, as an initial survey of other cell types with pacemaking activity or high-firing rates did not show the cell class–wide ISR activation that was characteristic of striatal CINs (73). It is intriguing to consider whether neuromodulatory neurons in particular may use the ISR differently than most cells because of greater challenges in maintaining cellular homeostasis due to frequent firing rates and extensive axonal arborizations relative to other neurons. To address this possibility, more work will be needed to functionally characterize the ISR activity state in specific neuromodulatory cell populations and determine whether there are others wherein “always stressed” (i.e., chronic ISR activation) is the norm.

Beyond being an unusual biochemical property, ISR activity in neuromodulatory neurons appears to play critical roles in the function of those neurons with downstream consequences on learning and memory. In striatal CINs, cell-specifically lowering ISR activity changed behavior in two types of learning paradigms: instrumental task learning in a lever pressing task and spatial learning and recall in the Morris water maze (73). Intriguingly, in the Morris water maze task, striatal CIN–specific experimental results closely mirror results reported in other studies using non-cell-selective pharmacological and genetic ISR-inhibiting manipulations (64, 77). These results raise the possibility that striatal CINs could be a cellular target for some of the cognitive effects that have been associated with ISR-inhibiting manipulations.

How widely are the behavioral effects of global ISR manipulations exerted through effects on neuromodulatory neurons? This depends, in part, on how many other neuromodulatory neurons share the functional property of the ISR being chronically activated. At least some nonstriatal populations of cholinergic neurons assessed by SPOTlight do not show the same high ISR state, but a comprehensive analysis of all cholinergic neurons has not been reported. In particular, it would be interesting to know whether basal forebrain cholinergic neurons share the high ISR state, since they broadly influence cognition and behavior through widely distributed cortical and subcortical projections.

Dopaminergic neurons are another cell class of interest. Like CINs, many dopaminergic neurons show tonic, pacemaking action potential firing, and the axons of both cell types are among the longest and most extensively arborized of any type of neuron, a feature that is regarded as an additional metabolic challenge to support (78). SPOTlight measures in dopamine neurons did not show uniform ISR elevation as in the case of striatal CINs, but there was a broad range, indicating the possibility that some dopamine cell subtypes might highly engage the ISR at steady state (73). Recent evidence supports at least that dopaminergic neurons significantly rely on the ISR for normal function. In dopamine neurons, cell-specific genetic manipulations of the genes for both PERK and the eIF2α phosphorylation site modify learning behavior (79).

Another well-established effect of ISR inhibition in the brain is that it modifies the threshold of experience that is needed to establish long-lasting memory and synaptic plasticity (9, 64). Synaptic plasticity is a protein synthesis–dependent process in which long-term changes in synaptic efficacy (or “strength”) arise as a result of neuronal activity/experience (80). In synaptic plasticity, there is a role for local protein synthesis at the synaptic site undergoing plasticity (81, 82). There is also evidence that protein synthesis related to synaptic plasticity may specifically include ISR-regulated protein synthesis. In hippocampal CA3 pyramidal neurons, levels of AMPAR subunits in dendrites, a measure for synaptic strength, inversely correlated with phospho-eIF2α levels. These findings support a mechanism by which ISR activation locally at synapses drives synaptic plasticity (65).

**Figure 4:**
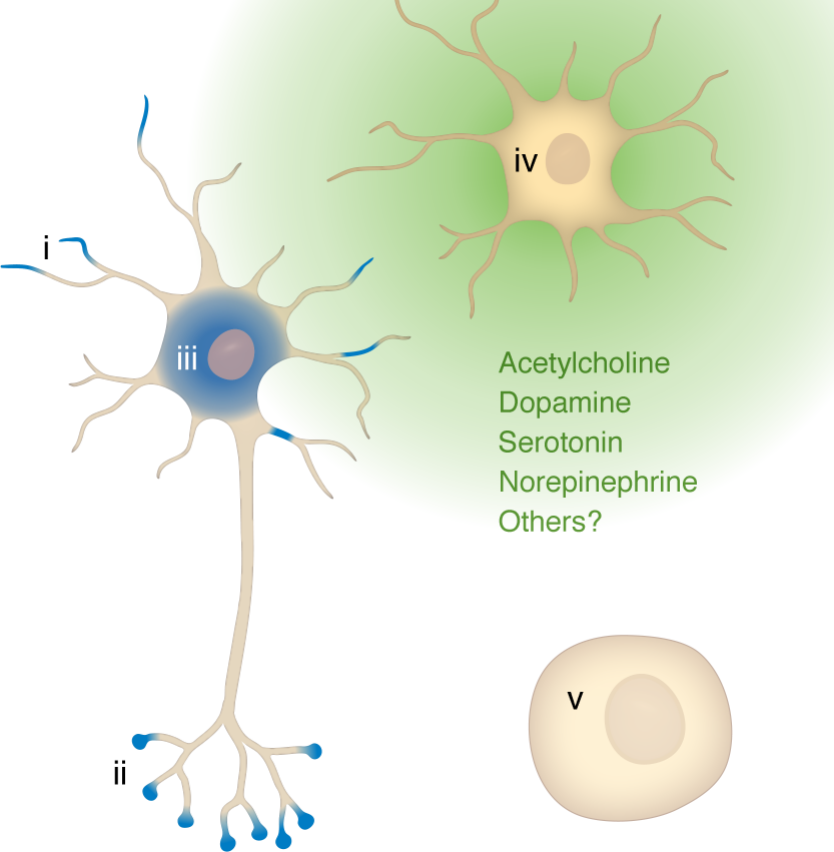
Model indicating potential sites where ISR protein synthesis may mediate its roles in synaptic plasticity, learning, and memory behavior. Blue shading indicates sites where (i) postsynaptic, (ii) presynaptic, or (iii) somatic regional protein synthesis may occur as a result of ISR activation in support of synaptic plasticity. (iv) Neuromodulatory neuron (producing acetylcholine, dopamine, serotonin, norepinephrine, or possibly other neuromodulators) that is distinct from a cell undergoing synaptic plasticity. (v) Other cell type contributors, as yet unidentified, which are also distinct from cells undergoing synaptic plasticity.

In light of recent findings that ISR manipulations restricted to neuromodulatory neurons are sufficient to modify learning and memory behavior (73, 79), it is worth considering whether the sites of action for the ISR in modifying synaptic plasticity may be broader than local action at synapses undergoing plasticity and include non-cell-autonomous effects via actions in neuromodulatory neurons. Both acetylcholine and dopamine are well known to influence the induction of long-term synaptic plasticity (75). Formally, there are a number of other sites for ISR actions to consider beyond local effects at synapses. ISR-related protein synthesis might influence learning and memory through modification of global protein synthesis in the cell undergoing plasticity, or through actions in another cell type, such as other neuromodulatory cells, glia, or oligodendroglia (Figure 4). Moreover, while some studies employing ISR manipulations used cell-specific manipulations (79, 87–89), many did not (64, 77, 83–86), leaving the corresponding site or sites of ISR action often uncertain.

Could ISR actions in neuromodulatory cells underlie the observed effects of systemic ISR manipulations on changing synaptic plasticity in other cells? A recent study deleting PERK supports this mechanism. Cell-specific deletion of PERK in dopaminergic neurons was sufficient to change the magnitude of synaptic plasticity induced at synapses between glutamate inputs and target cells in the striatum and hippocampus (i.e., not the manipulated dopamine neurons) (79). Both of these forms of synaptic plasticity were known to be influenced by dopamine signaling, and the investigators also showed that PERK deletion in dopamine neurons modified dopamine release. These results demonstrate that PERK deletion in dopamine neurons has non-cell-autonomous effects of influencing the magnitude of synaptic plasticity in other cell types.

The relationship between ISR actions in CINs and synaptic plasticity in other cells has not been established. However, it is noteworthy that cholinergic signaling is known to play a role in two forms of synaptic plasticity that have been shown to be sensitive to ISR inhibition. First, in the ventral tegmental area (VTA), a midbrain region with critical roles in reward processing, ISR inhibition facilitated a form of LTP at glutamatergic inputs to dopamine neurons that is induced by cocaine (90). Cholinergic signaling has also been shown to facilitate VTA LTP (91, 92). Second, ISR inhibition blocked a form of LTD at the glutamatergic synapses of cortical inputs to striatal projection neurons (5); this specific form of LTD is also inhibited if dopaminergic signaling in cholinergic neurons is impaired (67). Together, these observations raise the possibility that ISR actions in neuromodulatory neurons may contribute not only to behavioral learning effects but also to the underlying synaptic plasticity events. More targeted cell-specific perturbations are needed to fully appreciate the mechanisms by which the ISR influences synaptic plasticity and to disentangle the relative contributions of ISR acting in the cell undergoing plasticity from other supporting cells such as neuromodulatory cells, astrocytes (89), or even other cell types (Figure 4). With ISR modulation emerging as a clinical target for a variety of indications (9), it is all the more urgent to gain mechanistic clarity on the sites and mechanisms by which the ISR exerts it behavioral and plasticity effects.

## Lessons learned from dystonia, serendipity, and future directions

The biochemical pathway that constitutes the ISR plays a major role body-wide in proteostasis yet also has clear and potent brain-specific actions that influence synaptic plasticity and learning behavior in a variety of normal and diseased brain settings (9, 12). These features have made the ISR an attractive therapeutic target for both diseases directly attributed to ISR dysfunction, as well as a host of other conditions that may benefit from such a plasticity- or proteostasis-modifying inroad.

Intrigued by a clinical disorder in which movements associated with a highly learned skill, such as playing the piano, were selectively impaired, we suspected that brain learning mechanisms might be part of the pathophysiology. So, we set out on a path to understand the relationship between dystonia and synaptic plasticity. Along the way, dystonia models pointed us to the ISR. We learned that impairment of the ISR may be a common denominator process that leads to dystonia in various distinct genetic and sporadic forms of the disease (Figure 3). Looking ahead, this insight into dystonia mechanisms presents a number of potential strategies to target the ISR therapeutically. Success in this effort will depend on understanding how to optimally target the ISR, how many dystonias share the ISR mechanism, and how they can be identified.

Beyond understanding specific roles of the ISR in disease processes, this inquiry also led to a widened appreciation of how healthy cells engage the ISR and the ISR’s functional consequences in neuromodulatory cells with regard to synaptic plasticity and behavior. This Review has focused on emerging evidence within cholinergic and dopaminergic neurons that the ISR may need to be chronically active in those cell types to maintain the integrity of their normal function. ISR inhibition in these cell types remaps the relationships among neuromodulation, synaptic plasticity, and behavior.

These insights lead to a host of new questions. What other cells in the brain (and beyond) engage the ISR chronically to function normally? What factors regulate high ISR state in these cells? Can we learn about mechanisms by which cells escape ISR-induced apoptosis through these unusual cases? In diseases involving neuromodulatory cell dysfunction, is a basally high demand for ISR an Achilles’ heel contributing to their vulnerability?

The serendipity in science is reflected in our lab’s work on the ISR: exploring a relative diversion from our core interests in basal ganglia synaptic plasticity mechanisms ultimately led us back into the brain, opening a new view of how the ISR acts in the brain to influence learning and memory. Who would have guessed that our initial experiments with kidney cells in a petri dish (5) would lead to new basic understanding of how the brain works? The freedom to pursue high-risk, curiosity-driven work is crucial for the health and well-being of the biomedical research enterprise. As scientists and clinicians, we can all contribute to the work to educate about the frequently unpredictable paths from discovery to clinical impact and advocate for funding to support a broad base of scientific discovery research.

## Version 1 04/01/2024

Electronic publication

## Version 2 04/12/2024

corrected typographical errors
